# Trends and Insights from Transportation Congestion Pricing Policy Research: A Bibliometric Analysis

**DOI:** 10.3390/ijerph19127189

**Published:** 2022-06-11

**Authors:** Bhavna Singichetti, Adam Dodd, Jamie L. Conklin, Kristen Hassmiller Lich, Nasim S. Sabounchi, Rebecca B. Naumann

**Affiliations:** 1 Injury Prevention Research Center, University of North Carolina at Chapel Hill, 725 Martin Luther King Jr. Blvd., CB #7505, Chapel Hill, NC 27599, USA; rnaumann@email.unc.edu; 2 Department of Epidemiology, University of North Carolina at Chapel Hill, 725 Martin Luther King Jr. Blvd., CB #7505, Chapel Hill, NC 27599, USA; 3 Health Sciences Library, University of North Carolina at Chapel Hill, 335 S. Columbia St., CB #7585, Chapel Hill, NC 27599, USA; ddodd@email.unc.edu (A.D.); jconklin@unc.edu (J.L.C.); 4 Department of Health Policy and Management, University of North Carolina at Chapel Hill, 1105E McGavran-Greenberg Hall, CB #7411, Chapel Hill, NC 27599, USA; klich@unc.edu; 5 Department of Health Policy and Management, Center for Systems and Community Design, City University New York, Graduate School of Public Health and Health Policy, 55 West 125th St., New York, NY 10027, USA; nasim.sabounchi@sph.cuny.edu

**Keywords:** congestion pricing, travel demand management, transportation policy, cordon pricing, zone pricing, traffic congestion, transportation systems

## Abstract

Toll-based congestion pricing (CP) policies are increasingly implemented globally for alleviating road traffic congestion. Several interconnected factors affecting or induced by CP implementation include air quality/emissions, travel time, and road user safety. We sought to examine and characterize research output and patterns across several domains (e.g., health, policy acceptability) surrounding toll-based CP policies, in order to identify where research has focused and where gaps exist. We conducted a structured review and identified 2333 relevant publications, using semi-supervised and machine learning strategies combined with manual review. Annual publication counts peaked in 2015 (n = 122). Themes identified from title and abstract terms included policy implementation characteristics, advanced transportation modeling methods and approaches, and public perception and acceptability. Authorship networks indicated a lack of interdisciplinary research. Country analyses identified the US, China, and the UK as the most frequently represented countries, and underrepresentation from low-income countries. Findings indicate that research focused on specific road user types (e.g., pedestrians) and safety impacts, and equity considerations were relatively sparse compared to other topics (e.g., policy economics, public perception). Additional research on these critical topics is necessary to ensure that such policies are designed to promote positive and equitable effects on road user health and safety.

## 1. Introduction

### 1.1. Background

Congestion pricing (CP) is a growing international trend for mitigating road traffic congestion in major cities. CP policies are strategies that work by financially encouraging traffic shifts (e.g., to different times of day or different roadways, to other forms of transportation) that ease congestion in high traffic areas, particularly in urban centers [1]. Research indicates that CP policies can be effective at addressing congestion and generating revenue [1,2,3]. However, the impacts of CP policies reach far beyond congestion mitigation. Since transportation systems directly impact several aspects of individual health (including physical activity, air quality/emissions, injury, and safety), CP policies have the potential to make large impacts on several critical areas of public health. Further, the ability to access transportation to meet daily needs (e.g., obtain healthy food, access healthcare, travel to employment) is a critical determinant of health and well-being, and a CP policy’s role in supporting or hindering safe, healthy, and equitable transportation access deserves careful consideration when designing and implementing such policies.

While CP policies include a variety of approaches, toll-based policies (e.g., High Occupancy Toll (HOT) lanes) are the most widely used. Toll-based policies are generally grouped into the following categories: (1) variably-priced lanes (e.g., HOT lanes), (2) variable tolls on entire roadways (e.g., toll roads), (3) zone-based or cordon charges (charges to enter or drive within a certain region), and (4) area-wide or system-wide charges (per-mile or per-kilometer charge for travel within a certain region) [1,4]. When a city selects a policy, it is further tailored to its specific needs, environment, and political pressures (e.g., making exceptions for specific vehicle types), and a variety of potential impacts are considered and debated (e.g., revenue, equity, air quality).

### 1.2. Related Studies and Research Contribution

Providing a comprehensive examination of which CP policy facets have been studied, when, and by which countries can illuminate the current landscape of research for countries or cities considering initial implementation or refinement of their policies. Although systematic reviews of CP policies have been published, they have focused on specific aspects or consequences of policies in isolation (e.g., public acceptability) [5,6,7,8]. One recent review considered eight real scenarios where CP policies were proposed and identified four major factors that influenced public acceptability: invasion of privacy, uncertainty, difficulty of implementation, and equity [8]. Equity was the focus of two earlier reviews of CP policy literature, which examined literature to explore concerns of inequity (e.g., individuals with lower incomes potentially experiencing greater harm due to the burden from additional costs) and ways these policies may be more equitable than existing systems (e.g., those who contribute to congestion have to pay, reductions in air pollution) [6,7]. While these isolated reviews have enriched understanding of specific facets of policy implementation and impact, CP research could benefit from an overarching roadmap of the interconnected foci of publications, illuminating areas of high research productivity and gaps. This examination can highlight critical gaps in specific areas of CP policy research, which can guide targeted international research efforts to help inform future health and transportation policy.

Bibliometric analysis is an innovative and effective tool for examining the breadth of publications over time relevant to a focused research topic and can help identify patterns and trends in research output (e.g., sub-topics, geographic representation), [9] highlighting emerging research areas and those with a dearth of analyses. Bibliometric techniques have been used to summarize research output and identify patterns and gaps in several key areas (e.g., transportation research patterns and characteristics, COVID-19 research, economics of certain global regions, and mental health among specific subpopulations) [10,11,12,13,14]. To our knowledge, no recent structured review has taken a holistic and comprehensive perspective on CP policy research and the many domains it impacts (e.g., health, economic, safety, equity).

### 1.3. Research Objectives and Organization

The purpose of this study was to examine and characterize research output and patterns surrounding toll-based CP policies, using novel bibliometric methods, to inform future international research efforts. This paper is organized into the following sections: material and methods, results, discussion, and conclusions.

## 2. Materials and Methods

### 2.1. Search Strategy and Information Sources

To summarize and characterize the current and historical landscape of CP policy research, we conducted a structured search and bibliometric analysis. We searched the following transportation, health, and social science-related databases from their dates of inception through a final search date of 7 February 2021: Transport Research International Documentation (TRID), Web of Science, PubMed, and Scopus. TRID, which includes US and international research, is maintained by the Transportation Research Board (TRB) and sponsored primarily by state and federal (US) Departments of Transportation. TRID includes both publications from peer-reviewed journals and research reports. PubMed is a database of biomedical literature and is maintained at the US National Library of Medicine (NLM) and by the National Center for Biotechnology Information (NCBI), part of the National Institutes of Health (NIH). Web of Science and Scopus are large scholarly research databases that include peer-reviewed literature from around the world and across disciplines, including the sciences, social sciences, and humanities.

The search strategy for each database included many keywords describing CP policies, such as “congestion pricing”, “congestion fees”, “zone-based pricing”, “road user charges” and “toll schemes” (see full list in Table S1 in Supplementary Materials). Searches in each database were filtered by publication type to match our inclusion criteria of articles, reviews, and reports. No date limits were applied to the search. The complete, reproducible search strategy is available in the Supplementary Materials.

### 2.2. Inclusion and Exclusion Criteria

Publications were included if they (1) were either published in peer-reviewed journals or if they were published as reports; (2) contained, at minimum, an abstract or summary in English; and (3) examined a road or traffic-related CP toll-related policy. As defined by the US Federal Highway Administration, CP “is a way of harnessing the power of the market to reduce the waste associated with traffic congestion and works by shifting purely discretionary rush hour highway travel to other transportation modes or to off-peak periods…” [1]. Publications were required to have a pricing or charging (e.g., referred to costs, prices, charges) component and a traffic congestion component (e.g., referred to congestion alleviation, travel time reduction). Publications examining tradable electronic ticket/credit schemes, assuming all other features of the policy met the definition of a toll-related CP policy, were included. Publications that discussed structures and facilities without mention of a CP policy or were non-toll related (e.g., dynamic parking pricing) were excluded from this study. Publications were also excluded if they were in the format of news articles, project proposals, and books or book chapters.

### 2.3. Publication Selection

**Figure 1:**
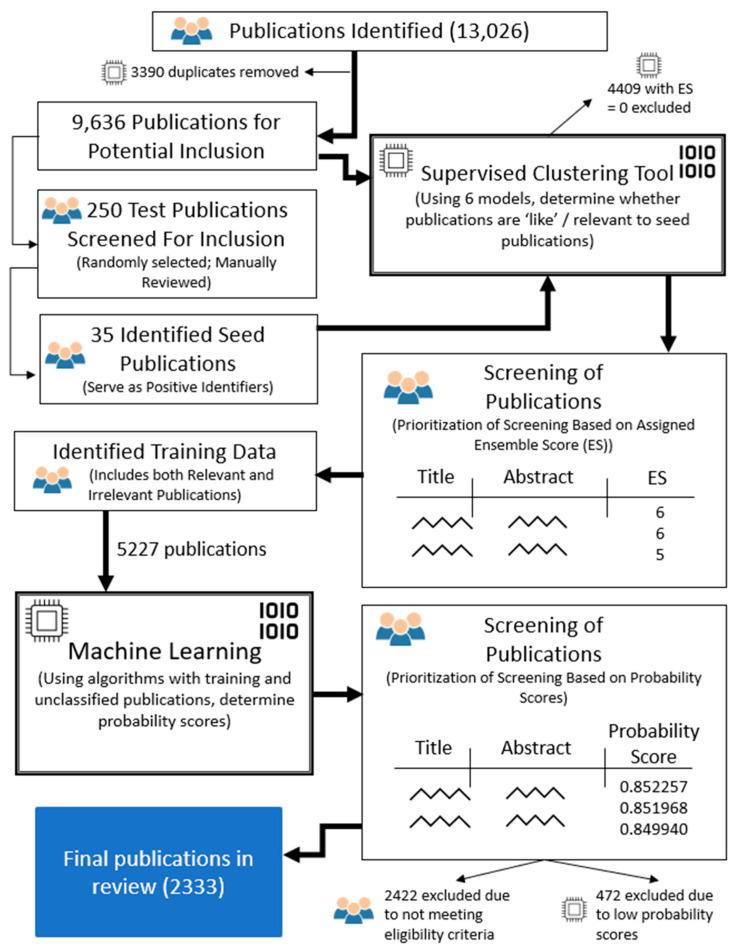
Flow diagram describing manual and automated steps in publication selection process.

The publication selection process included a series of manual and automated steps, described in Figure 1. Initial search results were imported into EndNote X8 (Philadelphia, PA, USA) and duplicates were removed. We used Covidence (Veritas Health Innovation, Melbourne, Australia, available at www.covidence.org, accessed 7 February 2021), an online screening tool that facilitates bibliometric and systematic reviews, to screen publications. We prioritized publications for manual screening using a two-phase, automated approach that relied on semi-supervised learning and machine learning. We conducted both phases using DoCTER software (Document Classification and Topic Extraction Resource) (ICF, Fairfax, VA, USA). DoCTER applies algorithms to text with publication titles and abstracts.

To complete the first phase, we imported a random sample of 250 publications into Covidence, and two researchers screened them to identify a set of known relevant publications, or “seed” publications. We identified 35 seed publications, which we then used for supervised clustering in which the remaining corpus of publications were grouped with the seed publications. Those clusters containing a higher number of seed publications are expected to be highly relevant [15]. DoCTER’s supervised clustering uses six clustering models based on two algorithms (K-means and non-negative matrix factorization) and three cluster sizes (10, 20 and 30). This approach then produces an ensemble score (ES) for each publication that ranges from zero to six and represents the number of models that found the publication to be relevant (with a score of zero being least relevant and a score of 6 being most relevant). We prioritized publications with ESs ranging from 3–6 for manual screening, and two authors screened these titles and abstracts (single screener per publication).

In the second phase, we used machine learning to prioritize those publications with an ES of 1–2 from the first phase. Publications with an ES of 0 were deemed irrelevant and removed from our analysis. We used a set of included and excluded publications from those publications we manually screened in the first phase for training data in this second phase. Through machine learning, DoCTOR provided a probability score for each publication, and we manually screened publications in Covidence with the highest probability scores in batches of 500 until relevance dropped off (probability score of 0.421).

### 2.4. Data Analysis and Visualization

Networks were constructed using the VOSviewer application (version 1.6.17, Centre for Science and Technology Studies, Leiden University, The Netherlands, accessed 13 August 2021), based on available data fields in the four databases. All publications were included in network maps describing the distribution of and relationships between key terms, and frequency of publications and collaborations between authors. Only those publications in Scopus were used to construct network maps based on authors’ country or territory (due to availability of this field). In all maps, size of each node represented the number of publications, and lines between and proximity of nodes reflect the relative connectivity (frequency of connection) between those nodes.

Key terms were used to evaluate topic networks. Terms were identified from the title and abstract fields of every publication. Authors reviewed identified terms, consolidated where appropriate (e.g., singular/plural forms, synonymous terminology), and removed non-topic terms (e.g., ‘etc.’, ‘i.e.,’). The resulting list was used to create the final visualizations, including a network map with common clusters of terms (for terms that appeared at least 10 times) and a map with a time overlay for identifying patterns of terms used over time. A similar strategy was used for authorship, where the list of authors was reviewed and consolidated where appropriate. The network map was used to identify author productivity and collaborations, and the map with a time overlay was used to identify patterns of authorship collaboration over time. Finally, the Scopus-only country maps, where ‘country’ was based on co-author countries or territories, were similarly developed to identify collaboration clusters and publications patterns over time. Interactive versions of all network maps included in this paper and the Supplementary Materials can be downloaded from the Carolina Digital Repository, an archive of scholarly content maintained by the University of North Carolina at Chapel Hill (https://cdr.lib.unc.edu/, accessed on 10 April 2022). Network maps can be viewed either in the VOSViewer desktop client or the VOSViewer web interface (https://app.vosviewer.com/, accessed on 10 April 2022).

## 3. Results

**Figure 2:**
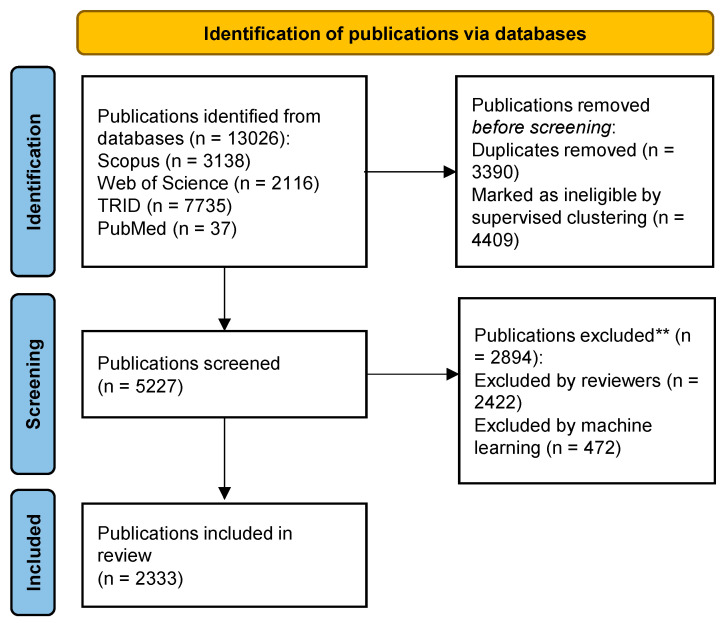
PRISMA Flow Diagram for Congestion Pricing Bibliometric Analysis *. * Modified for a bibliometric analysis with no screening of full-text publications. ** See Section 2.2 for description of inclusion and exclusion criteria.

The overall screening strategy followed Preferred Reporting Items for Systematic Reviews and Meta-Analysis (PRISMA [16]) guidelines, and is described in Figure 2. Additional details of this semi-automated approach are described in Section 2.3 and in Figure 1. A total of 13,026 relevant publications were identified from the four databases. Of these, 3390 duplicates were identified and removed, and 4409 were deemed ineligible by the supervised clustering method described previously (i.e., due to an extremely low probability score for relevance). Of the remaining 5227 publications included in the screening process, 2894 were excluded by either machine learning or by reviewers (based on the inclusion and exclusion criteria described in Section 2.2). A final 2333 publications were included in the bibliometric analysis.

The earliest publication included in the analysis was from 1956. The annual publication count remained under 20 until 1990 and was highest in 2015 (n = 122). About 87% of the publications (n = 2029) came from peer-reviewed journals, while the remainder were reports (n = 304). The five sources with the greatest number of relevant publications were Transportation Research Record, Transportation Research Part A, Transportation Research Part B, Transport Policy, and Transportation.

### 3.1. Title and Abstract Terms

**Figure 3:**
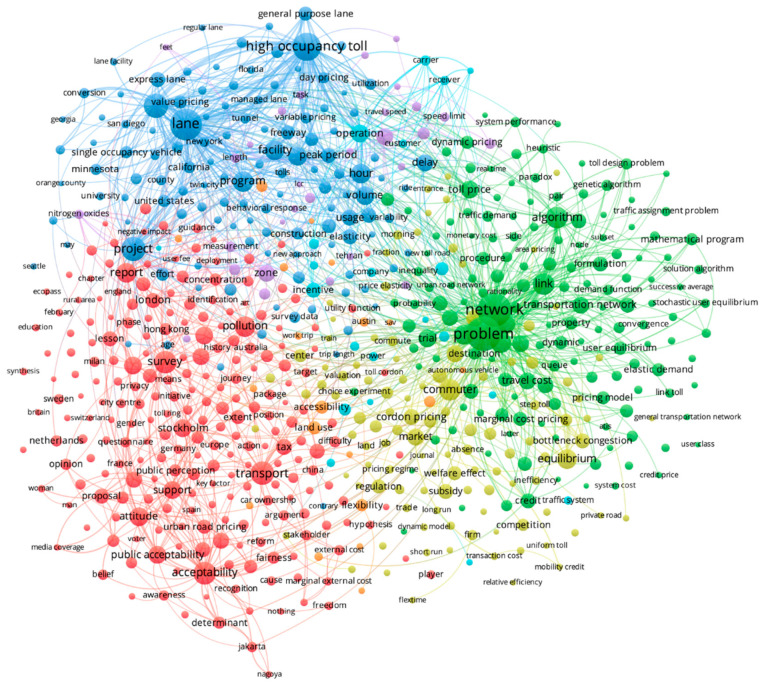
Title and abstract terms in congestion pricing bibliometric review.

Figure 3 shows clusters of terms commonly identified in titles and abstracts of the publications included in this review. The most common terms were ‘problem’ (n = 834), ‘network’ (n = 796), ‘lane’ (n = 666), and ‘high occupancy toll’ (n = 482). Seven clusters were identified using VOS clustering. The three largest clusters are distinguished by blue, green, and red in the figure. The blue cluster focuses on a range of structural implementation terms and policy types, including terms such as ‘high occupancy toll’, ‘facility’, ‘lane’ and ‘peak period’. The green cluster includes terms that focus on diverse transportation modeling methods, characteristics, and approaches, including ‘network’, ‘algorithm’, and ‘link’. The red cluster includes terms related to population perceptions of CP, such as ‘attitude’, ‘acceptability’, and ‘support’, and effects relevant to perception such as ‘pollution’ and ‘external costs.’ In the four smaller clusters, the terms that appeared most frequently included ‘commuter’ (n = 257), ‘equilibrium’ (n = 187), ‘speed’ (n = 136), ‘zone’ (n = 133), and ‘optimal road pricing’ (n = 123). A significant amount of overlap in the types of terms is observed in these clusters. Figure S1 (see Supplementary Materials) shows the same title and abstract terms network map with a time overlay, demonstrating a general shift in terminology from terms related to implementation in the early 2000s toward terms related to acceptability and advanced modeling approaches to study CP implementation after 2010.

### 3.2. Author/Research Collaboration Networks

**Figure 4:**
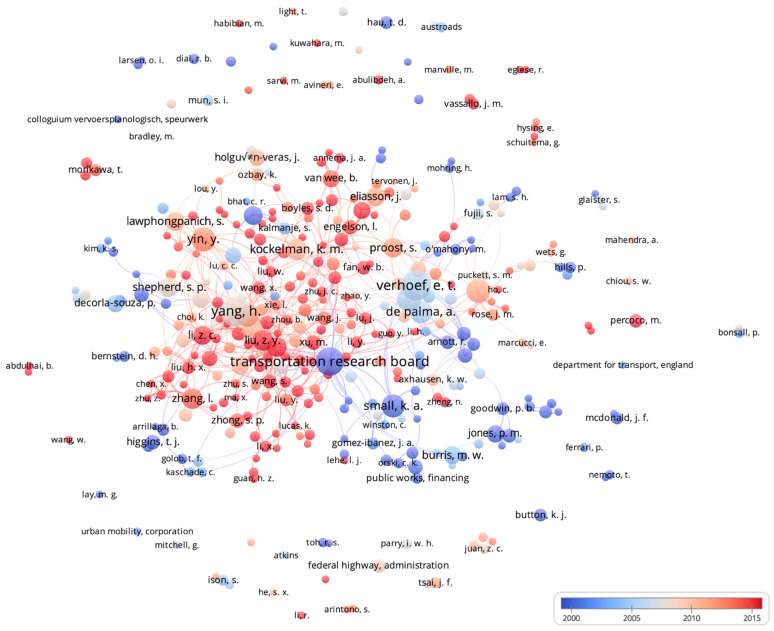
Author/Research Collaboration Networks in congestion pricing policy research with time overlay.

Collaboration networks for authors and research organizations from publications meeting a threshold of at least three publications are depicted in Figure 4. 362 distinct authors (individuals and research groups/institutes), and 79 clusters were identified based on publication collaborations. Author information is based on the ‘author’ field of publication citations; therefore, any individual, group, or organization credited with authorship in a citation was included in this analysis. Transportation Research Board was the most active contributor to CP literature among authors with an average publication year prior to 2000 (n = 37), followed by K.A. Small (n = 25), and the Texas Transportation Institute (n = 16). For average publication years between 2000 and 2004, the most active contributors were M.W. Burris (n = 15); P. DeCorla-Souza (n = 14); and H.S. Mahmassani (n = 12). Frequently published authors with average publication years 2005 and later included H. Yang (n = 46); E.T. Verhoef (n = 42); and Y. Yin (n = 28). Publications by Transportation Research Board and Texas Transportation Institute included introductions to and descriptions of CP policies, evaluations and case studies of existing CP policies, considerations for implementation (e.g., potential barriers such as political and public acceptability, planning and design issues), and implications of CP policies (e.g., air quality, energy use, equity). Publications by DeCorla-Souza also included descriptions of CP policies, as well as examinations of financial and economic aspects of implementing and maintaining CP policies (e.g., revenue), while Burris focused on toll price elasticity, public acceptability, and traveler choices in networks with CP policies. Small, Mahmassani, Yang, Verhoef, and Yin used advanced modelling and simulation approaches in many of their publications. Common topics for these five authors included assessments of time-varying tolls and dynamic pricing, strategies for optimization and efficiency with CP policies, analysis of driver choices in networks with CP policies, and public acceptability. Figure S2 (see Supplementary Materials) depicts collaboration clusters of authors and research organizations in this analysis.

### 3.3. Country Representation in Research

**Figure 5:**
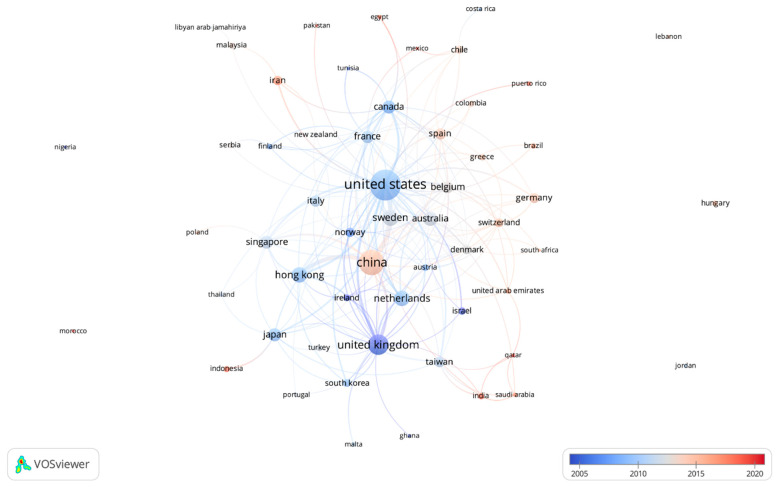
Country representation in congestion pricing policy research with time overlay.

Patterns in country or territory (described as ‘country’ in this analysis and based on co-author country/territory) representation were only available for publications indexed in Scopus (n = 1380) and are displayed with a time overlay in Figure 5. Overall, the top five countries represented in the literature were the US (n = 439), China (n = 265), the United Kingdom (n = 154), Sweden (n = 86), and Hong Kong (n = 78). The United Kingdom had an average publication year of 2005. By 2009, publication patterns shifted to include the US, the Netherlands (n = 77), Canada (n = 51), and Hong Kong. Sweden and Australia (n = 59) gained greater representation, on average, in late 2011 and early 2012, after which patterns shifted to include China, Iran (n = 22), Spain (n = 33), Germany (n = 23), and Switzerland (n = 17). Most recently, research representing countries such as Indonesia (n = 9), India (n = 7), Puerto Rico (n = 3) and Qatar (n = 2) have also started to appear in published research. Countries closer to the center of the map had high degrees of relational connectivity with their neighboring country nodes than the countries displayed along the outer edges of the map. For example, the US had an especially high degree of relational connectivity with Sweden, Australia, and Belgium—representing a frequent number of collaborations between authors from these countries. Meanwhile, countries such as Morocco, Jordan, Hungary, Nigeria, and Lebanon had no relational connectivity with other countries. Figure S3 (see Supplementary Materials) shows this network map with clusters based on co-author countries.

## 4. Discussion

We conducted a structured review and bibliometric analysis to understand the scope of existing literature on toll-based CP policies across several domains (e.g., health, economics, safety). The annual number of publications grew significantly between 1956 and 2015, with annual research output increasing from just 1 in 1956 to 122 in 2015. A wide variety of topic areas were studied, including CP implementation logistics, public perception and acceptability, and network algorithms and advanced modeling techniques. Analysis of authorship networks identified a large number of network clusters collaboratively studying a wide range of topics, including factors affecting implementation (e.g., design considerations, political acceptability, public perceptions), modelling/simulation approaches to understand network dynamics and examine specific features of CP policies (e.g., time-varying tolls), and impacts of CP policy implementation (e.g., traveler choices in networks with CP policies, air quality/emissions changes). Finally, analysis of country representation revealed notable shifts in research output across the globe as countries explored CP policy implementation, with early research productivity in the United Kingdom, moving to the US (and other countries such as the Netherlands, Canada, and Japan), and eventually countries such as China, Germany, and Iran. Overall, more and more countries were represented with each passing year.

Analysis of title and abstract terms identified patterns of research focus. We anticipated some terms would be highly prevalent, such as ‘network’, ‘high occupancy toll’, and ‘algorithm’ and found this to be the case. The high frequency of ‘network’ and ‘algorithm’ align with themes identified from publications by top contributors in this analysis, many of whom conducted multiple simulation-based studies to assess potential CP policy effects. The frequency of ‘high occupancy toll’ may reflect the prevalence of this policy in the US, which has the greatest publication representation in this study. Specifically, HOT lanes have been widely considered in the US due to their ease of structural implementation (existing High Occupancy Vehicle lanes converted to HOT lanes, or new HOT lanes added to existing freeways) and high levels of public support [1,17]. Notably, our analysis revealed research clusters focused on public acceptability and perception, which is often a key barrier to toll-based CP policy implementation [8,18,19].

Several topic gaps were identified in this analysis. First, CP policies can place a sudden, significant, and disproportionate burden on commuters, yet terms related to equity were only in a small proportion of titles and abstracts. For example, vehicle users may be faced with daily charges for a commute they may not be able to afford or may extend driving travel times that can burden not only vehicle users but also their families. Second, terms relevant to specific road user types and modes (e.g., pedestrian, motorcycle (ist), bicycle (ist)) and terms related to safety were extremely sparse in titles and abstracts, yet these are extremely important aspects of CP policies, which encourage travelers to change their route choices, commute patterns, and departure times. Individuals may shift to more affordable modes of transportation such as public transit and motorcycles (which are often exempt or have smaller congestion charges), or shift to non-motorized modes of transportation (e.g., walking, bicycling) [20]. Resulting changes in traffic flow, increased speeds, and individuals adjusting to new transportation modes may influence patterns of fatal and non-fatal injury for different road user types [21,22,23,24]. These gaps in research on CP policy implications for equity, safety, and specific road user types must be addressed as these factors are critical for successful planning and implementation of CP policies.

CP and other road safety strategies impact numerous domains (e.g., health, safety, economic) and as such will be most successful when designed and implemented with input from individuals with different areas of expertise, such as urban planning, engineering, politics/government, and public health. The analysis of authorship and collaboration networks highlighted the high productivity of not only individual researchers (often from academic institutions) but also major transportation organizations such as the Transportation Research Board and the Federal Highway Administration; yet few organizations from other fields were identified. New interdisciplinary perspectives could enrich future research on CP policies, particularly when addressing the previously mentioned gaps of equity, safety impacts, and specific road user types. Additionally, successful implementation of CP policies requires thoughtful consideration of community needs and evolving technology; however, publications describing community research as well as new technology (e.g., autonomous vehicles) were relatively lacking.

Analysis of countries represented in the publications from Scopus indicated that research output appeared to roughly align with CP policy planning and implementation. For example, London implemented their first CP policy in 2003 followed by policy modifications in later years, corresponding with an average publication year of 2005 for United Kingdom-based research output [2]. In Sweden, CP policies were implemented first in Stockholm (2007) and later in Gothenburg (2013), roughly corresponding to an average publication year of 2011 in our analysis [25,26]. Additionally, China has not implemented a CP policy to date, but such strategies have been evaluated for feasibility in more recent years [19,27,28], explaining the later average publication year. Overall, lower income countries were extremely underrepresented in all CP policy research, with the majority of research from these countries appearing only in most recent years. Although US publications included collaborations with numerous countries, the analysis of collaboration networks revealed that the US cluster did not include as many other countries as expected, indicating that the majority of research produced by authors based in the US did not include co-authors from other countries. The analysis of country collaborations and resulting network map did identify strong network connections between other groups of countries (clusters) including China, Hong Kong, and South Korea; Italy, Norway, Sweden, Australia, Denmark, and Austria; and Singapore, Japan, Poland, Thailand, Turkey, and Indonesia. Additional cross-discipline and inter-country collaborations could serve to advance the CP research field, filling important gaps, and ensuring lessons are shared so that future CP policy design and implementation builds from the evidence base.

### 4.1. Limitations

This study provided a comprehensive examination of research on toll-based CP policies by tapping into four large databases of scholarly literature with no limitations placed on date of publication. Few restrictions were placed on inclusion (i.e., included both peer-reviewed publications and reports). Still, the requirement that at least a summary or abstract of the article needed to be available in English may have resulted in the exclusion of topically relevant publications. Additionally, the screening approach was semi-automated and included use of novel machine learning techniques, which streamlined review of a large number of publications for inclusion in the bibliometric analysis. The analytic methods used in this study included network maps with cluster visualizations, which provided a unique way of looking at publication patterns and trends and the relationships that exist within the published literature. However, the types of patterns examined were restricted by field types available in the four databases. For example, the assessment of author/research collaboration networks was based on author fields of publication citations in each database, which is based on the citation of that publication and may include both individual authors and institutions. In particular, analysis of country representation and collaborations was restricted to those publications available in Scopus only and based on the country of the co-author affiliation, and therefore may not be representative of all publications in this study. Still, the analyses conducted provide valuable insights into the magnitude and distribution of toll-based CP literature. Next, the methods used in this study were designed to give a comprehensive picture of existing toll-based CP literature; therefore, in our analysis of title and abstract terms, we conservatively eliminated only terms that objectively provide no useful information, such as ‘and’, ‘the’, and ‘but.’ Finally, inclusion of non-toll-based CP policies (e.g., parking pricing) was beyond the scope of this review and analysis.

### 4.2. Future Research Recommendations

Cities and countries considering CP policies are currently predominantly using toll-based policies; however, future reviews should explore emerging research on non-toll-based policies. While this study sought to understand the breadth of existing toll-based CP literature, future research may focus on specific sub-topics, particularly those that address research gaps identified by this study. Future research should also include multi-disciplinary and international collaborations to assess specific impacts of CP policies on different road user types, particularly vulnerable road users (e.g., pedestrians, bicyclists), examine the safety impacts of these policies, and evaluate whether the impacts of these policies are equitable for all individuals affected by CP policy.

## 5. Conclusions

CP policies have been developed, evaluated, and implemented as a method for managing increased urban traffic congestion. A large body of research, spanning multiple decades, has been published on toll-based policies, as evidenced by the number and distribution of publications identified and analyzed in this study. Findings indicate that relatively low proportions of existing CP publications address equity and health impacts. These aspects are critical for a number of health (e.g., physical activity, air quality), safety (e.g., injury prevention), and ethical (e.g., access to daily needs) reasons, and they play an important role in public acceptability—which can serve as a key facilitator or barrier to successful policy implementation. Future research efforts should leverage multidisciplinary expertise and prioritize addressing these gaps to support evidence-based policy implementation in urban city centers.

## Supplementary Materials

The following supporting information can be downloaded at: https://www.mdpi.com/article/10.3390/ijerph19127189/s1, Table S1: Detailed Search Strategy, Figure S1: Title and abstract terms with time overlay, Figure S2: Author and research collaboration network clusters, Figure S3: Country collaboration networks.
