# Supplementary material for: Trends and Insights from Transportation Congestion Pricing Policy Research: A Bibliometric Analysis
Source: Int J Environ Res Public Health. 2022 Jun 11;19(12):7189. doi: 10.3390/ijerph19127189 (PMC9222647; doi:10.3390/ijerph19127189)
Supplement: Supplementary file 1 [file ijerph-19-07189-s001.zip › ijerph-1700448-supplementary.pdf]

SUPPLEMENTARY MATERIAL

Table S1. Detailed Search Strategy

Set #	Search Terms
	Scopus
1	TITLE-ABS-KEY (("congestion pricing" OR "congestion prices" OR "congestion price" OR "congestion charging" OR "congestion charges" OR "congestion charge" OR "congestion tax" OR "congestion taxes" OR "congestion taxing" OR "congestion fee" OR "congestion fees" OR “congestion toll*” OR "congestion subsidy" OR "congestion subsidies" OR “congestion policy” OR “congestion policies” OR “congestion strategy” OR “congestion strategies” OR “congestion zone” OR “congestion zones” OR cordon OR “Zone-based pricing” OR “zonal-based pricing” OR “Zone-based charg*” OR “zonal-based charg*” OR “zone pricing” OR “zone charg*” OR “zonal charg*” OR “zonal scheme” OR “zonal schemes” OR “zone scheme” OR “zone schemes” OR “distance-based pricing” OR “area-based pricing” OR “area-wide charg*” OR “area-based charg*” OR “per-mile pricing” OR “per-mile charg*” OR “network pricing” OR “mileage-based user fee” OR “mileage-based user fees” OR “entry-based pricing” OR “variable pricing lane” OR “price managed lane” OR “price managed lanes”) AND (traffic OR transportation OR road OR roads OR highway OR highways OR automobile OR automobiles OR car OR cars OR vehicle OR vehicles))
2	TITLE-ABS-KEY (“road pricing” OR “road value pricing” OR “road charging” OR “road charge” OR “road charges” OR “road user charge” OR “road user charges” OR “road use charge” OR “road use charges” OR “road use charging”)
3	TITLE-ABS-KEY ((“toll scheme” OR “toll schemes” OR “tolling scheme” OR “tolling scheme” OR “distance-based toll” OR “distance-based tolls” OR “distance-based tolling” OR “variable toll” OR “variable tolls” OR “variable tolling” OR “dynamic toll” OR “dynamic tolls” OR “dynamic tolling”) OR ((tolls OR tolling OR "high occupancy vehicle lane" OR "high occupancy vehicle lanes") AND (traffic OR road OR roads OR highway OR highways OR automobile OR automobiles OR car OR cars OR vehicle OR vehicles) AND (congestion OR congested OR “transportation demand management” OR “traffic demand management”)))
4	#1 OR #2 OR #3
5	AND (LIMIT-TO (DOCTYPE , "ar") OR LIMIT-TO (DOCTYPE , "re") OR LIMIT-TO (DOCTYPE , "rp"))
	Web of Science
1	TS=(("congestion pricing" OR "congestion prices" OR "congestion price" OR "congestion charging" OR "congestion charges" OR "congestion charge" OR "congestion tax" OR "congestion taxes" OR "congestion taxing" OR "congestion fee" OR "congestion fees" OR “congestion toll*” OR "congestion subsidy" OR "congestion subsidies" OR “congestion policy” OR “congestion policies” OR “congestion strategy” OR “congestion strategies” OR “congestion zone” OR “congestion zones” OR cordon OR “Zone-based pricing” OR “zonal-based pricing” OR “Zone-based charg*” OR “zonal-based charg*” OR “zone pricing” OR “zone charg*” OR “zonal charg*” OR “zonal scheme” OR “zonal schemes” OR “zone scheme” OR “zone schemes” OR “distance-based pricing” OR “area-based pricing” OR “area-wide charg*” OR “area-based charg*” OR “per-mile pricing” OR “per-mile charg*” OR “network pricing” OR “mileage-based user fee” OR “mileage-based user fees” OR “entry-based pricing” OR “variable pricing lane” OR “price managed lane” OR “price managed lanes”) AND (traffic OR transportation OR road OR roads OR highway OR highways OR automobile OR automobiles OR car OR cars OR vehicle OR vehicles))
2	TS=(“road pricing” OR “road value pricing” OR “road charging” OR “road charge” OR “road charges” OR “road user charge” OR “road user charges” OR “road use charge” OR “road use charges” OR “road use charging”)
3	TS=((“toll scheme” OR “toll schemes” OR “tolling scheme” OR “tolling scheme” OR “distance-based toll” OR “distance-based tolls” OR “distance-based tolling” OR “variable toll” OR “variable tolls” OR “variable tolling” OR “dynamic toll” OR “dynamic tolls” OR “dynamic tolling”) OR ((tolls OR tolling OR "high occupancy vehicle lane" OR "high occupancy vehicle lanes") AND (traffic OR road OR roads OR highway OR highways OR automobile OR automobiles OR car OR cars OR vehicle OR vehicles) AND (congestion OR congested OR “transportation demand management” OR “traffic demand management”)))
4	#1 OR #2 OR #3
5	AND Refined by: DOCUMENT TYPES: (ARTICLE OR EARLY ACCESS)
	TRID
1	("congestion pricing" OR "congestion prices" OR "congestion price" OR "congestion charging" OR "congestion charges" OR "congestion charge" OR "congestion tax" OR "congestion taxes" OR "congestion taxing" OR "congestion fee" OR "congestion fees" OR “congestion toll*” OR "congestion subsidy" OR "congestion subsidies" OR “congestion policy” OR “congestion policies” OR “congestion strategy” OR “congestion strategies” OR “congestion zone” OR “congestion zones” OR cordon OR “Zone-based pricing” OR “zonal-based pricing” OR “Zone-based charg*” OR “zonal-based charg*” OR “zone pricing” OR “zone charg*” OR “zonal charg*” OR “zonal scheme” OR “zonal schemes” OR “zone scheme” OR “zone schemes” OR “distance-based pricing” OR “area-based pricing” OR “area-wide charg*” OR “area-based charg*” OR “per-mile pricing” OR “per-mile charg*” OR “network pricing” OR “mileage-based user fee” OR “mileage-based user fees” OR

	"entry-based pricing" OR "variable pricing lane" OR "price managed lane" OR "price managed lanes") AND (traffic OR transportation OR road OR roads OR highway OR highways OR automobile OR automobiles OR car OR cars OR vehicle OR vehicles)
2	"road pricing" OR "road value pricing" OR "road charging" OR "road charge" OR "road charges" OR "road user charge" OR "road user charges" OR "road use charge" OR "road use charges" OR "road use charging"
3	("toll scheme" OR "toll schemes" OR "tolling scheme" OR "tolling scheme" OR "distance-based toll" OR "distance-based tolls" OR "distance-based tolling" OR "variable toll" OR "variable tolls" OR "variable tolling" OR "dynamic toll" OR "dynamic tolls" OR "dynamic tolling") OR ((tolls OR tolling OR "high occupancy vehicle lane" OR "high occupancy vehicle lanes") AND (traffic OR road OR roads OR highway OR highways OR automobile OR automobiles OR car OR cars OR vehicle OR vehicles) AND (congestion OR congested OR "transportation demand management" OR "traffic demand management"))
4	#1 OR #2 OR #3
5	#4 AND Limit to Journal Articles, Reports, and Serials
PubMed	
1	(("congestion pricing"[tiab] OR "congestion prices"[tiab] OR "congestion price"[tiab] OR "congestion charging"[tiab] OR "congestion charges"[tiab] OR "congestion charge"[tiab] OR "congestion tax"[tiab] OR "congestion taxes"[tiab] OR "congestion taxing"[tiab] OR "congestion fee"[tiab] OR "congestion fees"[tiab] OR "congestion toll"[tiab] OR "congestion tolls"[tiab] OR "congestion tolling"[tiab] OR "congestion subsidy"[tiab] OR "congestion subsidies"[tiab] OR "congestion policy"[tiab] OR "congestion policies"[tiab] OR "congestion strategy"[tiab] OR "congestion strategies"[tiab] OR "congestion zone"[tiab] OR "congestion zones"[tiab] OR cordon[tiab] OR "Zone-based pricing"[tiab] OR "zonal-based pricing"[tiab] OR "Zone-based charging[tiab]" OR "Zone-based charges[tiab]" OR "Zone-based charge"[tiab] OR "zonal-based charging"[tiab] OR "zonal-based charges"[tiab] OR "zonal-based charge"[tiab] OR "zone pricing"[tiab] OR "zone charging"[tiab] OR "zone charges"[tiab] OR "zone charge"[tiab] OR "zonal charging"[tiab] OR "zonal charges"[tiab] OR "zonal charge"[tiab] OR "zonal scheme"[tiab] OR "zonal schemes"[tiab] OR "zone scheme"[tiab] OR "zone schemes"[tiab] OR "distance-based pricing"[tiab] OR "area-based pricing"[tiab] OR "area-wide charging"[tiab] OR "area-wide charges"[tiab] OR "area-wide charge"[tiab] OR "area-based charging"[tiab] OR "area-based charges"[tiab] OR "area-based charge"[tiab] OR "per-mile pricing"[tiab] OR "per-mile charging"[tiab] OR "per-mile charges"[tiab] OR "per-mile charge"[tiab] OR "network pricing"[tiab] OR "mileage-based user fee"[tiab] OR "mileage-based user fees"[tiab] OR "entry-based pricing"[tiab] OR "variable pricing lane"[tiab] OR "price managed lane"[tiab] OR "price managed lanes"[tiab]) AND ("Transportation"[Mesh:NoExp] OR "Motor Vehicles"[Mesh] OR traffic[tiab] OR transportation[tiab] OR road[tiab] OR roads[tiab] OR highway[tiab] OR highways[tiab] OR automobile[tiab] OR automobiles[tiab] OR car[tiab] OR cars[tiab] OR vehicle[tiab] OR vehicles[tiab]))
2	("road pricing"[tiab] OR "road value pricing"[tiab] OR "road charging"[tiab] OR "road charge"[tiab] OR "road charges"[tiab] OR "road user charge"[tiab] OR "road user charges"[tiab] OR "road use charge"[tiab] OR "road use charges"[tiab] OR "road use charging"[tiab])
3	("toll scheme"[tiab] OR "toll schemes"[tiab] OR "tolling scheme"[tiab] OR "tolling scheme"[tiab] OR "distance-based toll"[tiab] OR "distance-based tolls"[tiab] OR "distance-based tolling"[tiab] OR "variable toll"[tiab] OR "variable tolls"[tiab] OR "variable tolling"[tiab] OR "dynamic toll"[tiab] OR "dynamic tolls"[tiab] OR "dynamic tolling"[tiab]) OR ((toll[tiab] OR tolls[tiab] OR tolling[tiab] OR "high occupancy vehicle lane"[tiab] OR "high occupancy vehicle lanes"[tiab]) AND ("Motor Vehicles"[Mesh] OR traffic[tiab] OR road[tiab] OR roads[tiab] OR highway[tiab] OR highways[tiab] OR automobile[tiab] OR automobiles[tiab] OR car[tiab] OR cars[tiab] OR vehicle[tiab] OR vehicles[tiab]) AND (congestion[tiab] OR congested[tiab] OR "transportation demand management"[tiab] OR "traffic demand management"[tiab]))
4	#1 OR #2 OR #3

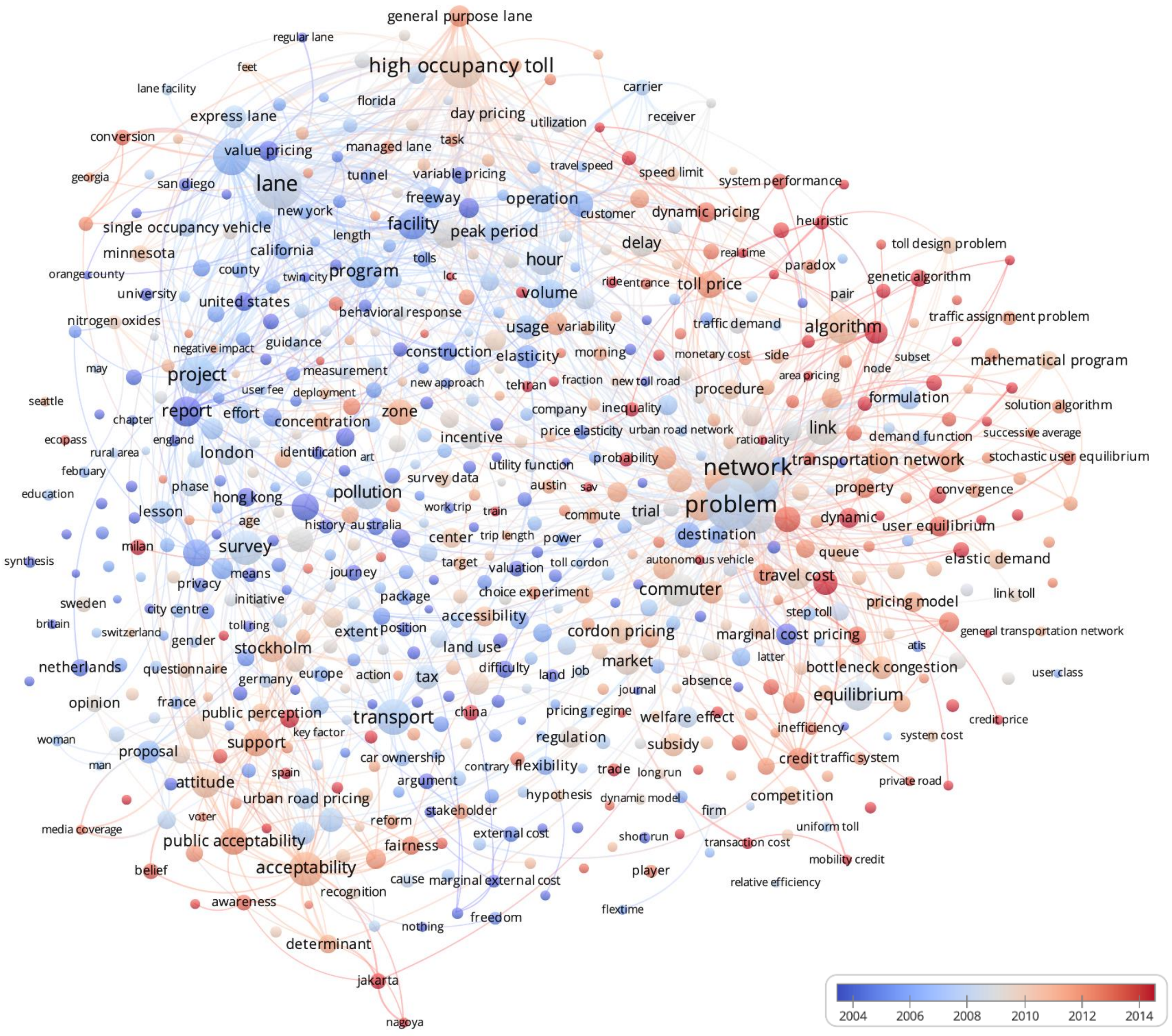

Figure S1. Title and abstract terms with time overlay

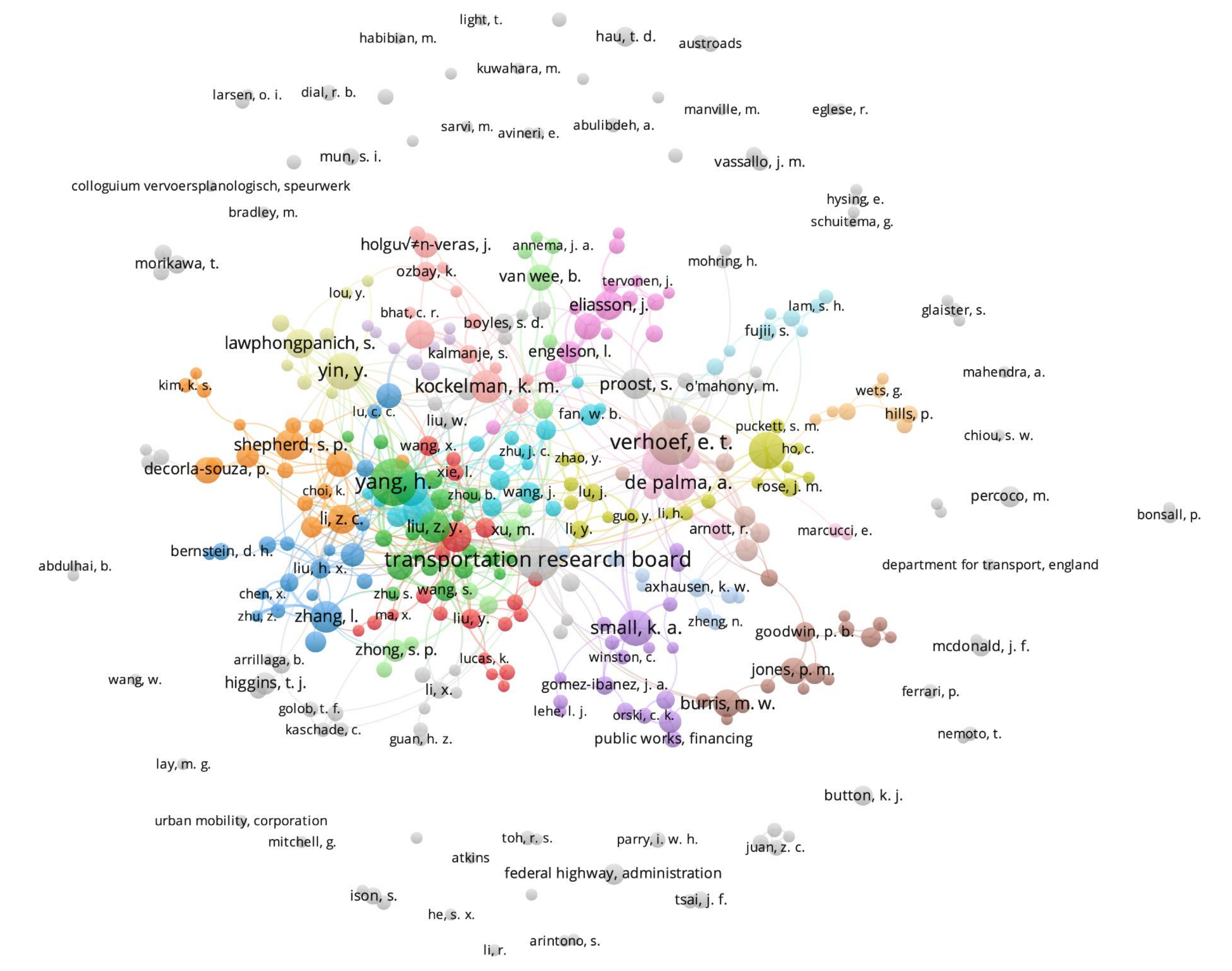

Figure S2. Author and research collaboration network clusters

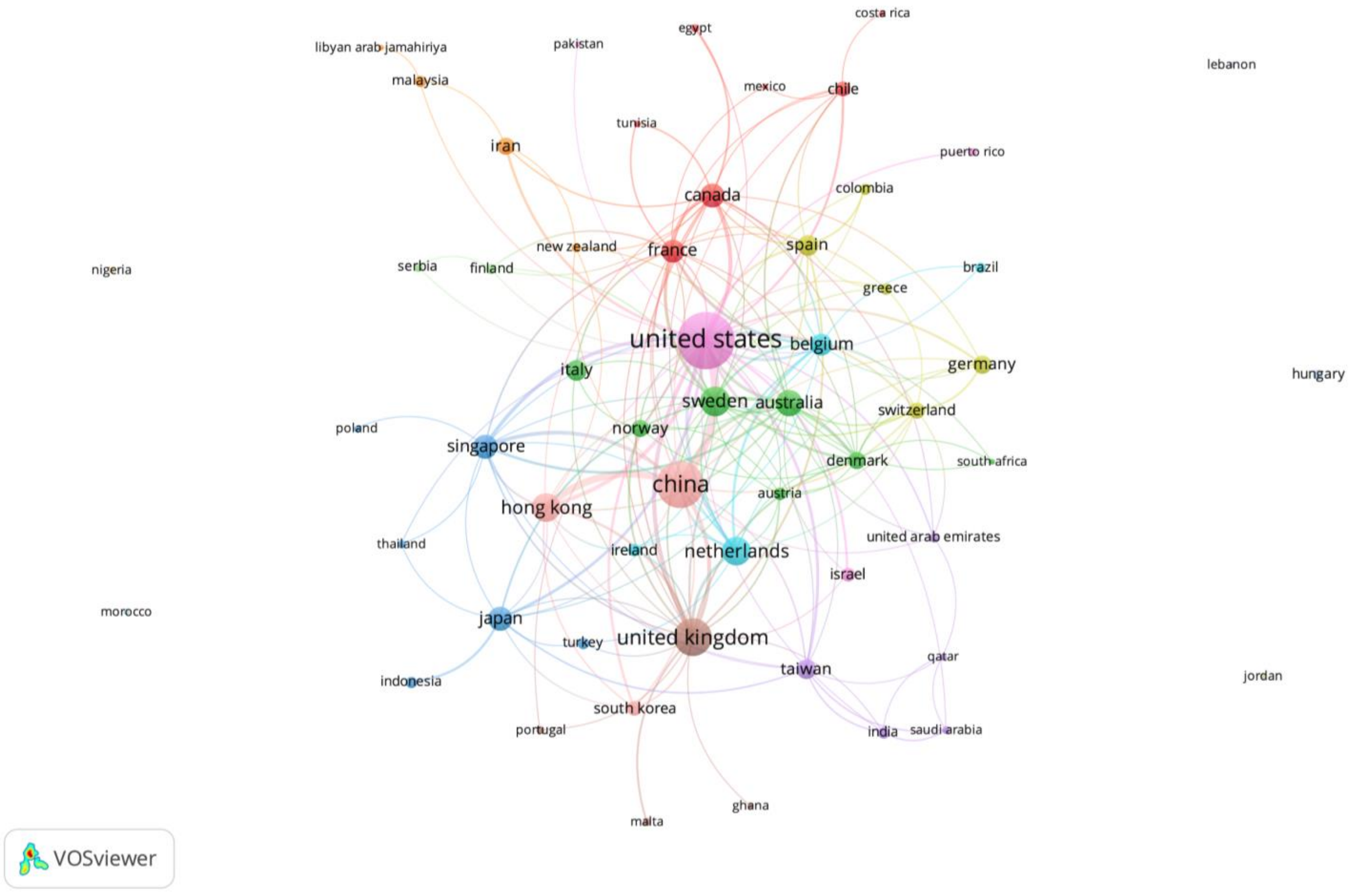

Figure S3. Country collaboration networks
